# Preparation and Test of NH_3_ Gas Sensor Based on Single-Layer Graphene Film

**DOI:** 10.3390/mi11110965

**Published:** 2020-10-28

**Authors:** Ting Liang, Ruifang Liu, Cheng Lei, Kai Wang, Zhiqiang Li, Yongwei Li

**Affiliations:** 1 Science and Technology on Electronic Test & Measurement Laboratory, North University of China, Taiyuan 030051, China; lrfnuc@163.com (R.L.); leicheng@nuc.edu.cn (C.L.); lizhiqiangnuc@163.com (Z.L.); liyongwei27@163.com (Y.L.); 2 Tianjin Weijia Environmental Technology Co., Ltd., Tianjin 300450, China; wangkaisdsh@163.com

**Keywords:** single-layer graphene, interdigitated electrode (IDE), wet transfer technology, ammonia detection, repeatability and stability

## Abstract

The ammonia sensing properties of single-layer graphene synthesized by chemical vapor deposition (CVD) were studied. The Au interdigitated electrode (IDE) was prepared by microelectromechanical systems (MEMS) technology, and then, the single-layer graphene was transferred to the IDE by wet transfer technology. Raman spectroscopy was used to monitor the quality of graphene films transferred to SiO_2_/Si substrates. Moreover, the theory of graphene’s adsorption of gases is explained. The results show that gas sensing characteristics such as response/recovery time and response are related to the target gas, gas concentration, test temperature, and so on. In the stability test, the difference between the maximum resistance and the minimum resistance of the device is 1 ohm without ammonia, the change is less than 1% of its initial resistance, and the repeatability is up to 98.58%. Therefore, the sensor prepared with high quality single-layer graphene has good repeatability and stability for ammonia detection.

## 1. Introduction

Ammonia is a colorless toxic gas with a pungent odor, which is harmful to human health. Studies have shown that people working with ammonia for a long time are often poisoned [1], and exposure to 25 ppm of NH_3_ can cause skin, eye, and lung irritation [2,3]. In addition, as a metabolite in human life, ammonia gas has been used as an important indicator in the diagnosis of diseases such as diabetes, kidney disease, malignant tumors, and lung cancer. Therefore, whether in the field of air quality detection or medical health, real-time detection of ammonia is essential.

According to the sensors category, commonly used ammonia detectors can be divided into infrared ammonia detectors, semiconductor ammonia detectors, and electrochemical ammonia detectors [4]. Resistance-type semiconductor ammonia detectors have wide application prospects due to their low cost, high sensitivity, and fast response. At present, most resistive semiconductor gas sensors have been reported to use metal oxides such as zinc oxide, tin dioxide, and tungsten oxide as gas-sensitive materials [5,6,7,8]. However, in practical applications, on the one hand, metal oxides need to be heated to a higher temperature to separate out ions such as O^2−^, O_2_^−^, and O^−^, thus achieving gas detection [9,10]; on the other hand, the device’s stability after repeated heating significantly reduces its detection limit [11,12]. Therefore, its development has been greatly limited. In 2004, two scientists at the University of Manchester in the United Kingdom first isolated graphene from graphite using a micromechanical method [13]. This is a kind of two-dimensional carbon nanomaterial composed of carbon atoms with sp^2^ hybrid orbitals. The carrier mobility of single-layer graphene at room temperature is about 15,000 cm^2^/(V·s) [14,15], with a large specific surface area [16,17]. In addition, all atoms are exposed to environmental conditions and small changes in carrier concentration can cause significant changes in graphene’s surface conductivity, which makes it ideal for gas detection [13]. Recently, studies have shown that single-layer graphene can detect gas molecules at the ppm level [18].

Many graphene-based sensors have been developed through doping, chemical modification, and other methods to detect NH_3_ response [19,20,21]. An effective method is that metal oxide is introduced to modify the original graphene and its derivatives [22,23]. The graphene’s sensitivity is improved after modification. However, due to some modification of the graphene channel created by the external elements, the sensor’s performance may be affected, which can cause certain electrical instability [24].

Based on the literature mentioned above, a single-layer graphene film was selected as the ammonia gas sensor’s sensitive material in this study. The single-layer graphene was transferred to the interdigitated electrode (IDE) prepared by the microelectromechanical systems (MEMS) process through wet transfer technology [25,26]. The responses of resistive graphene gas sensors to different temperatures and concentrations of ammonia were measured. In addition, the mechanism of graphene’s adsorption of ammonia gas was explained using the first principle. The experimental results show that the sensor prepared by this method has good stability and repeatability.

## 2. Materials and Methods

### 2.1. Fabrication of Devices

**Figure 1:**
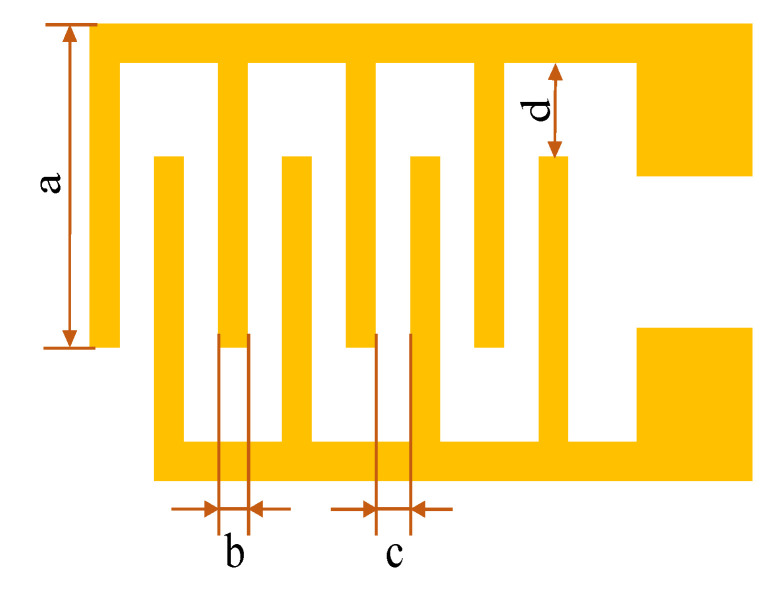
The interdigitated electrode.

The IDE is used as the sensor’s resistance. It has a planar structure, in which metallic conductors are placed in a comb-like arrangement. It is reported that the IDE can effectively reduce the test resistance of the gas sensing film, which is beneficial for testing the performance of the thin film’s gas sensing [27]. It has been widely used in thin film-type gas sensors [28]. However, the length, width, and distance between the fingers affect the resistance of the IDE [29]. As shown in Figure 1, in this article, we chose a = 500 μm, b = 500 μm, c = 500 μm, and d = 6000 μm, and the quantity of the IDE was 4; a is the length of IDE, b is the width of IDE, c is the distance between the two fingers, and d is the distance from the fork to the IDE’s edge.

**Figure 2:**
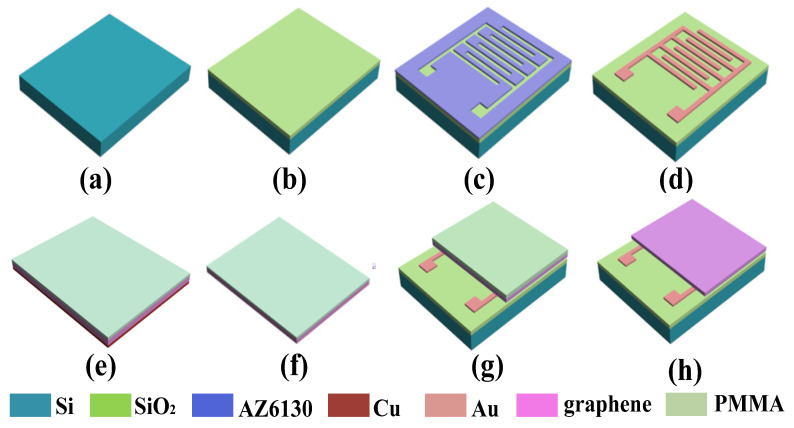
The processing flow of the interdigitated electrode (IDE): (**a**) Prepare the silicon wafer. (**b**) The deposition of SiO_2_ insulator layer by the plasma-enhanced chemical vapor deposition (PECVD). (**c**) The IDE structures were patterned by lithography. (**d**) Metal leads deposition (Ti 200 Å/Au 2000 Å) by magnetron sputtering, and the metal layer on AZ6130 photoresist was stripped in acetone by lift-off technology. (**e**–**h**): Single-layer graphene transferred onto the IDE cell by wet transfer technology.

The IDE was prepared using a standard microelectromechanical systems (MEMS) process, as shown in Figure 2. The critical process includes the following steps: the deposition of the SiO_2_ insulator layer, metal leads deposition, metal lift-off technology, and so on.

Firstly, the silicon wafers were cleaned using a standard inorganic cleaning process. The silicon wafers were cleaned in a strong acid mixture of sulfuric acid (hydrogen peroxide = 3:1 at 150 °C for 15 min, and then, in a strong alkali mixture of water: hydrogen peroxide: ammonia = 7:2:1 at 60 °C for 5 min). Next, the silicon wafers were rinsed with de-ionized (DI) water and blow-dried with nitrogen. The external contamination on the surface of the silicon wafer was removed in the above cleaning process. Then, a SiO_2_ film of 2000 Å thickness was deposited on the cleaned silicon wafer by plasma-enhanced chemical vapor deposition (PECVD), used as the insulating layer between metal electrode and silicon wafer, and the next process was lithography. Before photolithography, in order to increase the adhesion between the photoresist and the insulation layer, it was necessary to carry out Hexamethyldisiloxane (HMDS) steps on the wafer in an environment of 130 °C, and then, spin the positive photoresist AZ6130 on the silicon wafer at a speed of 3000 rad/min. The exposure technology adopted contact UV exposure and then, placed the wafer into the developer (DI water: AZ400K = 4:1), keeping for 30 s to form the electrode pattern. Before metal modification, oxygen plasma was used for surface activation treatment to increase the cleanliness and surface activation of silicon wafers. The parameters were as follows: power of 200 W, time of 2 min, and the carrier gas was oxygen. Furthermore, Ti 200 Å/Au 2000 Å metal layer was deposited by EXPLORED (a magnetron sputtering machine; Denton Vacuum LLC, Denton, TX, USA), and the parameters were as follows: vacuum degree of 5 × 10^−6^ torr, power of 500 W, and Ar with a flow rate of 14 sccm (standard cubic centimeter per minute). Upon the metal layer, the photoresist was dissolved in acetone solution, which is called lift-off technology. Then, the IDE cells were formed by ultraviolet laser fine processing equipment FPC03 (Delon laser, JiangSu, China). Finally, the monolayer graphene was transferred to the IDE by wet transfer technology.

The single-layer graphene used in this experiment was grown on a copper substrate by chemical vapor deposition technology, and PMMA was used as a protective layer, as shown in Figure 2e. Firstly, the cut graphene on copper substrate was put in the etching solution of copper foil (FeCl3, 1 mol/L), and after etching for 30 min, the copper foil was etched clean, and then, the etching solution on the surface was washed with deionized water; the etching result is shown in Figure 2f. The graphene/protective film was slowly released into deionized water, then the graphene was transferred to the surface of the IDE (Figure 2g). After that, it was dried at room temperature for 20 min and at 70 °C for 30 min to remove surface moisture, then cooled to room temperature. The above completed the transfer of graphene. To fully remove the PMMA layer, two boxes of acetone solution were prepared, the sample was immersed in one acetone solution for 10 min, and then, it was transferred to the other solution for 30 min.

### 2.2. Characterization of the Single Graphene

**Figure 3:**
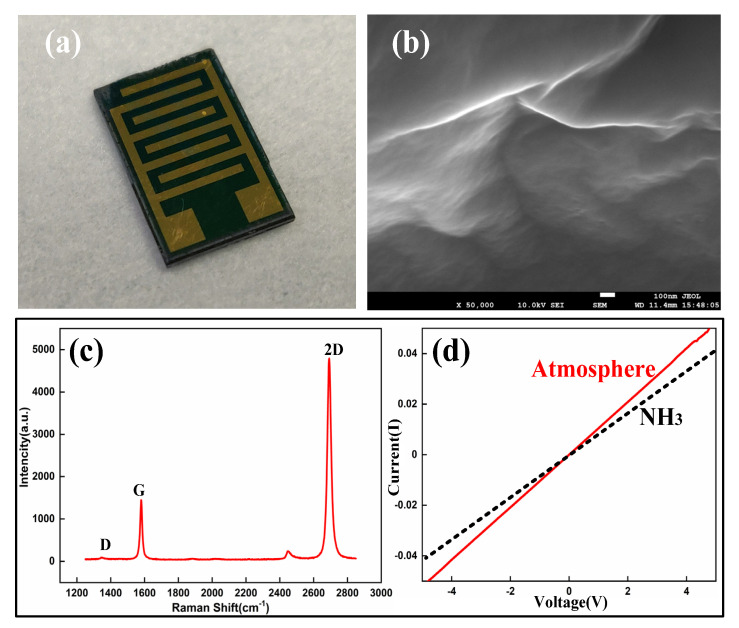
Single-layer graphene transferred onto a Si/SiO_2_ substrate. (**a**) Picture of the device, (**b**) the SEM of graphene, (**c**) the Raman spectra of single layer graphene, and (**d**) the I–V curves of metal and graphene ohmic contact.

As shown in Figure 3a–c, the graphene films transferred to Si/SiO_2_ substrates were characterized by Scanning Electron Microscopy (SEM) and Raman spectroscopy. Figure 3a is the physical picture of the device and Figure 3b is an SEM image of graphene film. It can be seen from the figure that the single-layer graphene is not a flat plane because single-layer graphene is a fragile material, which will lead to folds in the process of transfer. The Raman spectrum of the sample was obtained using a 514 nm laser with a spot size of about 2 μm. It can be seen from the picture that the spectrum is mainly composed of Raman peaks corresponding to the G band (1579 cm^−1^) and 2D band (2692 cm^−1^) [30], and the 2D peaks of the graphene are sharp and symmetrical with a perfect Lorentzian. The value of the full width at half maximum (FWHM) for the 2D peak is 14.19 cm^−1^, for the G peak is 26.79 cm^−1^, and the residual doping is 90.72 meV [31]. Figure 3c shows that the peak intensity ratio of the 2D band to the G band is greater than one, which is common for one-layer graphene [32,33]. Simultaneously, a semiconductor analyzer (Keithley 4200-SCS, Cleveland, OH, USA) was used to test the output characteristics (I–V) of the single-layer graphene-based sensor. As shown in Figure 3d, the output characteristics showed linear behavior in both negative and positive biased voltages; the two lines, respectively, represent the output characteristic curves in ammonia gas and atmospheric environments. It can be seen that the resistance in the ammonia gas environment is greater than the resistance characteristic in air. The experimental results clearly indicated that there was an ohmic contact between the metal electrode and graphene.

### 2.3. Experimental Test Platform

**Figure 4:**
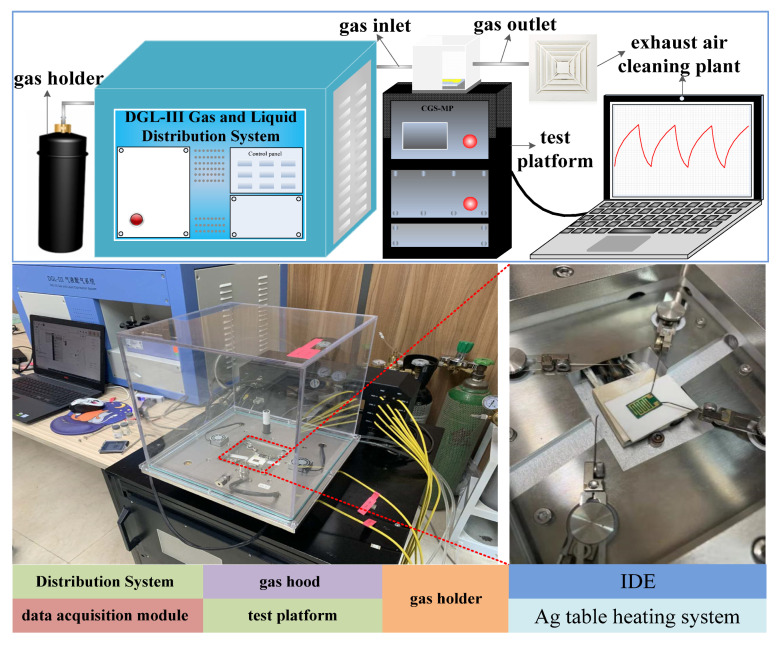
Photoelectric comprehensive test platform (include IDE, Ag table heating system, gas hood, intelligent distribution system, and data acquisition module).

Detection of the NH_3_ was performed in a photoelectric comprehensive test platform (CGS-MT). It is a closed device with a volume of 2000 cm^3^, the experimental device is shown in Figure 4 In the test, a microprobe was used to connect the signal electrode to the interdigital electrode. At the same time, it also matches an Ag table heating system, which can accurately control the temperature of the device. In this study, the dry air served as the carrier gas to mimic the practical detection environment as far as possible. In each test cycle, dry air was first introduced into the closed cavity for 10 min, and then, a constant concentration of NH_3_ was maintained for 10 min. This is a complete test cycle. In order to ensure there were no accidental experimental errors, three tests were performed in each group of experiments. In the experiment, the change of the device’s resistance was tested under different ammonia concentrations and different temperatures. We can also directly monitor device resistance changes under different conditions through external software tests in real-time.

## 3. Results and Discussion

The gas sensing properties of graphene were studied by measuring the resistance when they passed through gases in different environments. The sensor response was defined as the ratio of the change in ammonia resistance to in air, and was calculated by the following relation. Here, the *R_NH_3__* is the resistance of the sensor when exposed to ammonia gas, and the *R_air_* is the resistance of sensor when exposed to air [34].

$$S(\%)=\frac{R_{NH_{3}}-R_{air}}{R_{air}}\times 100\%$$

### 3.1. The Adsorption Mechanism of Graphene

The theory of graphene’s adsorption of gases is explained by the first principle, which is essentially the transferring of electrons between graphene and gas molecules [35]. As NH_3_ is a reducing gas, when exposed to NH_3_, the NH_3_ molecules come into contact with the single-layer graphene films and the electrons would be transferred from the NH_3_ molecules to the surface of graphene [23,35,36,37]. On the surface of graphene, electrons and holes can be recombined, thus causing the concentration of holes on the surface of the graphene to drop, which will reduce the conductivity of graphene [36,37,38]. Finally, it expressed as sensor resistance increased. Upon interrupting the NH_3_ supply, the NH_3_ molecules adsorbed on the graphene surface will be separated from graphene [23,35,36,37]. After NH_3_ desorption to the electron molecules, there will be no electron transfer on the graphene surface, and the resistance value will finally return to the initial state.

### 3.2. Humidity Measurement of the Sensor

**Figure 5:**
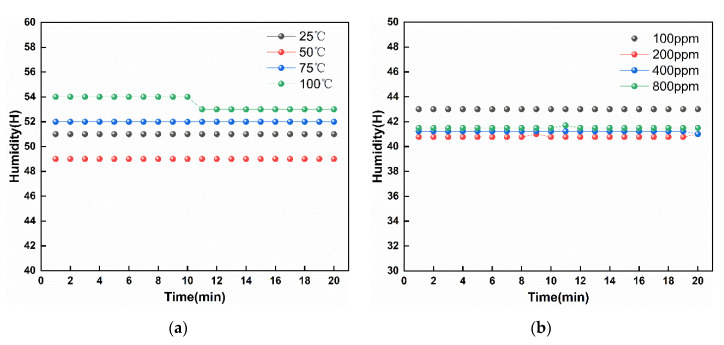
Humidity response of the sensor. (**a**) Response of sensor humidity over time in different temperature environments. (**b**) Response of sensor humidity over time in environments with different NH_3_ gas concentrations.

As we know, the graphene has a non-ignorable resistance response to the humidity [39,40]. In the experiments, we must ensure that each experiment environment’s humidity sensor is relatively stable to ensure the test results’ accuracy and reliability. Therefore, we chose the photoelectric comprehensive test platform (CGS-MT) for the test platform. It can realize humidity monitoring. Figure 5a shows an ammonia concentration at 100 ppm and a corresponding humidity sensor in different temperature environment values. In the four different ambient temperatures of 25, 50, 75, and 100 °C, the maximum fork finger of humidity is only 5% RH, which is a relatively small value. Moreover, in each ambient temperature, humidity is a relatively stable value. Similarly, we also tested the sensor’s humidity value when the ambient temperature was room temperature, and ammonia concentration was different. The experimental results are shown in Figure 5b. As shown in Figure 5b, at different ammonia concentrations, the maximum value of sensor humidity change is 3% RH. Therefore, in the subsequent tests, the sensor resistance changes are mainly caused by the ambient temperature and the sensor gas concentration.

### 3.3. The Response of Sensor

**Figure 6:**
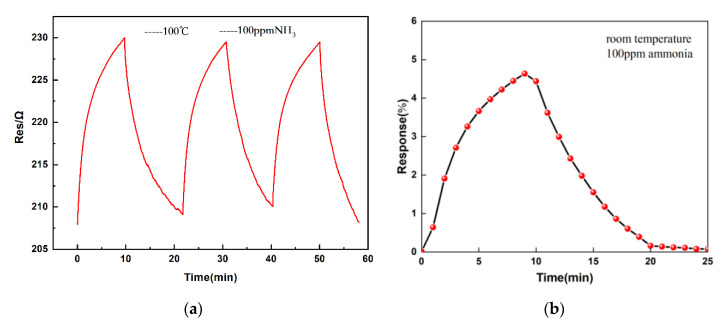
Response of the IDE for 100 ppm of NH_3_ gas at 25 °C. (**a**) The resistance of the sensor with time. (**b**) The response of the sensor.

Figure 6a shows the resistance of the sensor with time. This experiment was conducted at room temperature with NH_3_ concentration of 100 ppm. From Figure 6a, we found that the IDE’s resistance increased upon the exposure to NH_3_ gas. As shown in Figure 6b, we found that the maximum sensitivity was around 5.63% when exposed to 100 ppm NH_3_ for 5 min and the sensitivity of the sensor recovered to its baseline after 15 min. Meanwhile, with the reaction process’ occurrence, the sensor’s sensitivity was improved, but the reaction rate gradually decreased continuously. In the initial reaction stage, the change of sensor relative resistance is the largest. In the process of the reaction of 1–5 min, the response was 3.26%, and in response to the 5–10 min reaction stage, the resistance changes by only 1.37%. This can be attributed to the fact that the graphene surface is constant when exposed to NH_3_ and many ammonia molecules adsorbed to the graphene membrane surface. At the same time, electrons and holes can be recombined on the graphene membrane surface, causing the concentration of holes on the surface of the graphene to drop, causing the increase in sensor resistance. However, because the graphene surface adsorption of gas molecules is limited, as the reaction progresses, many gas molecules are adsorbed on the surface, the effective adsorption surface area decreases, and the response rate is slowed down. In the last, it kept a state of dynamic balance. After closing the intake channel, the dynamic balance was instantly broken, and the adsorbed gas molecules on the surface of the graphene film gradually decreased. At the same time, the surface electron migration rate attenuated, resulting in a decrease in resistivity, and finally, kept the stability.

### 3.4. The Concentrations Response of the Sensor

**Figure 7:**
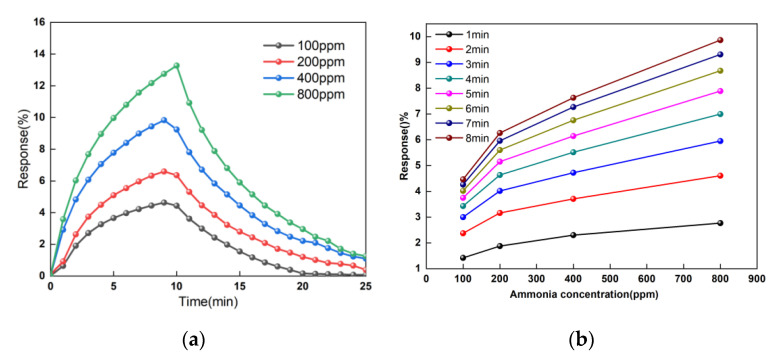
The concentrations response of the sensor. (**a**) The response of the sensor with time. (**b**) The response of the sensors with NH_3_ gas concentration.

In addition, we performed a series of tests at different gas concentrations. The experiment was carried out at room temperature, and the whole test process only changed the gas concentration. As shown in Figure 7a, we can find the higher the NH_3_ gas concentration is, the higher the sensor’s response speed and sensitivity will be. This is because NH_3_ gas with a higher concentration has more molecules per unit volume. When the intake channel is opened, more molecules are absorbed onto the surface of the graphene film. A large number of electrons provided by ammonia molecules compound with holes on the graphene surface, resulting in a decrease in hole concentration on the graphene surface. The decrease in cavity concentration leads to the decrease in the film surface’s conductivity, which is finally manifested as the increase in sensor resistance. Figure 7b is a graph of sensitivity changing with gas concentration. The eight curves represent the response of different time periods. From Figure 7b, the reaction to the sensor’s sensitivity with the increase in ammonia concentration in the environment increases. In different periods, the reaction of the overall trends is similar. In the 100–200 ppm ammonia environment, the sensor sensitivity change is higher than the sensor sensitivity value change in the 200–800 ppm environment. In theory, the gas concentration and the responsivity should present a relationship between the linear changes. However, the adsorption capacity of the ammonia gas molecules on the graphene surface is limited. With the increase in concentration, the adsorption does not exhibit linear properties, and the reaction speed of the graphene adsorption surface will reduce.

### 3.5. The Temperature Response of the Sensor

**Figure 8:**
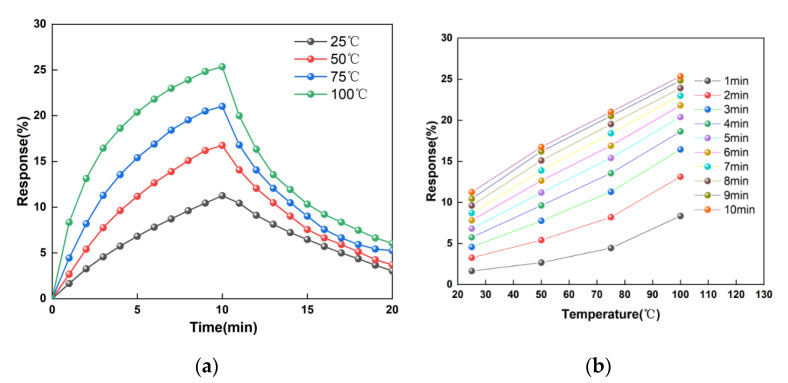
The temperature response of the sensor. (**a**) The response of the sensor with time. (**b**) The response of the sensors with different temperature.

The motion of molecules is related to temperature. For NH_3_, it causes the NH_3_ molecules to move violently at high temperatures. Therefore, the influence of temperature on the performance of the sensor is studied. In this paper, the corresponding tests are carried out at different temperatures. Figure 8a shows the real-time response curves at 25, 50, 75, and 100 °C with the 100 ppm ammonia environment. At the same concentration of gas in the environment, the sensor’s sensitivity increases with the temperature. The higher the temperature, the faster the thermal motion of the ammonia molecules. Simultaneously, the more molecular adsorption to the graphene membrane surface results in a constant reaction between holes on the graphene’s surface and electrons provided by the molecules of ammonia gas. Figure 8b is a graph of the sensitivity changing with temperature. The relationship between temperature and response presents a linear change, and as the reaction progresses, the linearity becomes higher and higher. In the initial stage of the reaction where molecules are in an active state, the increase in temperature and molecular thermal motion is more violent. Thus, the response sensitivity phenomenon in the high-temperature condition is higher than in the low-temperature condition. However, as the reaction tends to dynamic balance, in the 4 to 10 min stage of reaction, temperature sensitivity presents a linear state.

### 3.6. Repeatability and Stability Tests of the Sensor

**Figure 9:**
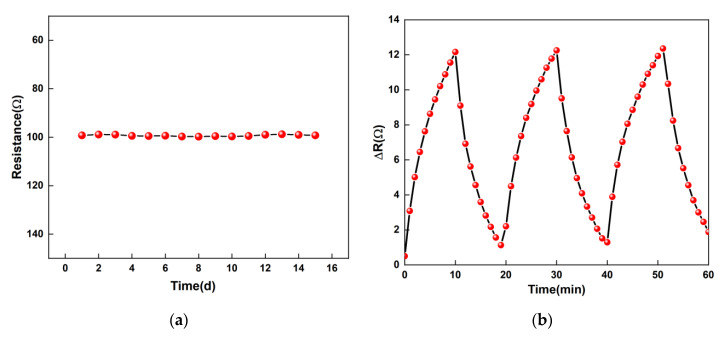
The repeatability and stability of the sensor. (**a**) The stability of the sensor with time. (**b**) The repeatability of the sensors with time.

Repeatability and stability are important parameters to characterize a sensor. Stability is the ability of a device to maintain the same performance over time. In this experiment, three IDEs processed in the same batch were selected for testing, and their test results were very similar. A graphical representation of the stability test results is shown in Figure 9a. The IDE has placed the sensor in the test environment’s stability to test the sensor’s stability. Through continuous observation of the IDE’s resistance for 15 days, the resistance is relatively stable and the sensor has good stability. The maximum resistance and the minimum resistance value of the difference are only 1 Ω, and the change is less than 1% of its initial resistance. Repeatability characterizes the degree of inconsistencies in each measurement of the same gas concentration measured by a sensor. Repeatability is an important indicator to determine whether a sensor can be used multiple times. Figure 9b studied inlet and exhaust repeatability randomly selected with ammonia concentration at 100 ppm and ambient temperature at 75 °C. It can be seen from the figure that the variation trend of the three tests is stable, each cycle is the same, and the fitting results show that the repeatability reaches 98.58%.

## 4. Conclusions

In this paper, a resistive graphene-based gas sensor with good repeatability and stability has been prepared, which is different from previous studies focusing on improving sensitivity and selectivity. Single-layer graphene was transferred to an IDE by wet transfer technology. Raman spectral analysis showed that monolayer graphene had fewer defects and stable performance. The experimental results show that the sensor prepared by this method has a good response to ammonia gas, and the response has a stable relationship with the ambient temperature and gas concentration when the ambient humidity is relatively stable. Through repeated tests, it is found that the stability error of the sensor is within 1%, and the repeatability reaches 98.58%. In the future, we will study the feasibility of graphene sensors for environmental monitoring and perform surface functionalization of single-layer graphene layers, based on which the sensor’s sensitivity will be improved.
